# Effectiveness of an edutainment video teaching standard precautions – a randomized controlled evaluation study

**DOI:** 10.1186/s13756-019-0531-5

**Published:** 2019-05-22

**Authors:** Aline Wolfensberger, Alexia Anagnostopoulos, Lauren Clack, Marie-Theres Meier, Stefan P. Kuster, Hugo Sax

**Affiliations:** Division of Infectious Diseases and Hospital Epidemiology, University Hospital Zurich, University of Zurich, Rämistrasse 100, CH-8091 Zurich, Switzerland

**Keywords:** Standard precautions, Universal precautions, E-learning, Online learning, Education, Edutainment, Infection prevention and control (IPC)

## Abstract

**Background:**

Standard precautions are essential to prevent pathogen transmission and nosocomial infections. We assessed learning effect (primary outcome) and satisfaction (secondary outcome) of watching a 5-min humorous “edutainment (=education and entertainment) video” on Standard Precautions compared to reading a written standard operating procedure (SOP) or receiving no intervention.

**Methods:**

This randomized controlled trial was executed at the University Hospital Zurich, Switzerland, a tertiary care centre with a state-of-the-art infection prevention programme. Healthcare providers (HCPs) of different medical departments were 1:1:1 randomized to watching the edutainment video (video group), reading the SOP (SOP group), or no study-specific intervention (no-intervention group). Online questionnaires included a knowledge assessment about Standard Precautions at time point (TP) 1 immediately after intervention, TP2 after 1 month, and TP3 after 3 months. Information about HCPs’ satisfaction with the learning method was collected. Variables were assessed within and between groups using the appropriate non-parametric tests. Predictors for knowledge of Standard Precautions were assessed by uni- and multivariable linear regression.

**Results:**

Overall, 363 predominantly female (78.2%) HCPs were included. At TP 1 and TP3, the video group scored better on the knowledge assessment against both the SOP and the no-intervention group (TP1 *p* < .001 and 0.001, TP3 *p* = 0.036 and 0.048). In the multivariable analysis, being member of the video group was an independent predictor for better knowledge scores. The video was rated higher than the SOP regarding satisfaction with learning experience, and video group participants more frequently indicated they would recommend their learning method to colleagues.

**Conclusions:**

Watching an edutainment video proved to be more effective to improve knowledge about Standard Precautions compared to reading an SOP or no intervention. Satisfaction with the learning method was superior in the video group, suggesting higher potential for future uptake.

**Electronic supplementary material:**

The online version of this article (10.1186/s13756-019-0531-5) contains supplementary material, which is available to authorized users.

## Background

Standard precautions are designed to ensure safety for healthcare providers (HCPs), patients and visitors [1]. They are essential to prevent transmission of pathogens that may be involved in hospital acquired infections. According to the Centers for Disease Control and Prevention (CDC), the following topics are listed as Standard Precautions elements: hand hygiene, personal protective equipment, respiratory hygiene/cough etiquette, patient placement, cleaning and disinfection of patient care equipment and instruments/devices, handling of textiles and laundry, safe injection practices, and handling of needles and other sharps [1].

Knowledge about Standard Precautions among HCPs is oftentimes limited [2–4], and the level of knowledge can vary substantially among institutions. It was found to be lower in long-term care and psychiatric institutions compared to acute care hospitals [3]. Also, knowledge and familiarity with Standard Precautions differs between HCPs, with nurses being more familiar with Standard Precautions than medical doctors [3]. Therefore, education and teaching of Standard Precautions across all healthcare institutions and all professions is crucial when infection prevention and control (IPC) practices are aimed to be successfully implemented.

In recent years, online learning (e-learning) and internet were used as an important resource for education, and became a progressively growing part of HCP education. E-learning – defined as educational intervention mediated electronically via the Internet [5] – has been shown to be associated with remarkably positive effects on learning performance compared to no intervention and with a similar learning effectiveness compared to traditional methods [6, 7]. An important advantage of e-learning is its flexibility [8]; it is easily accessible, available around the clock, and can be accessed and performed independently and repetitively – an important aspect considering the tendency of knowledge to fade over time. E-learning is cost-efficient; once established, it is a time- and resource-saving approach compared to traditional learning techniques [9]. E-learning techniques have been increasingly applied in the past and have shown positive results regarding the promotion of IPC topics in general [10–12], for hand hygiene [13, 14], and for prevention of hospital acquired infections [15, 16].

The IPC team of the University Hospital Zurich, Switzerland, created an educational video clip to improve HCP knowledge about Standard Precautions with the help of a professional film-making staff. As it is well known that emotions help learners to focus and facilitate uptake of information into long-term memory [17], we chose humor as the central emotional feature in this project. Positive emotions and the power of laughter can enhance the learning experience, and humor improves student performance by attracting and sustaining attention, reducing anxiety, enhancing participation, and increasing motivation [18]. As safety in aviation and healthcare are often compared [19], we decided to produce a mash-up between an in-flight safety video and infection prevention instructions. The final product was a 5 min “edutainment” video, combining education and entertainment.

Kirkpatrick proposed a “four-level model” for evaluating training programs [20]: ‘Reactions’ (level 1), assessing participant satisfaction; ‘Learning’ (level 2), assessing knowledge or skills of participants; ‘Behaviors’ (level 3), measuring performance in actual practice; and ‘Results’ (level 4), assessing the impact of education on the system, such as changes in patients’ health. Before launching the video, we conducted a randomized controlled trial to evaluate the educational impact (level 1) and user satisfaction (level 2) of our edutainment video compared to education by reading a standard operating procedure (SOP) about Standard Precautions or no intervention.

## Methods

### Study setting and participants

The study was conducted at the University Hospital Zurich, Zurich, Switzerland, a 950-bed tertiary-care teaching hospital covering all medical specialties except pediatrics and orthopedics. The University Hospital Zurich has 7200 employees, and around 67% thereof are working in patient care. We invited all hospital ward managers, heads of operating room personnel and therapists, as well as heads of several medical departments, to participate in the study with their entire team of HCPs, both registered and in training. We enrolled the teams based on the order of their replies and considering all professions to be represented. Ethical approval for this quality assessment and improvement project with anonymous data collection was not necessary according to the Swiss law on research on humans.

### Study design

Using a computer-generated code, we randomly assigned the participants in a 1:1:1 ratio to either watch the video (video group), to read the SOP (SOP group) - both study tasks called *intervention* from here on - or no specific task (no-intervention group). Participants in all three groups answered a questionnaire at three time points: At time point (TP1) right after the intervention in December 2016, 1 month later at time point 2 (TP2) in January 2017, and 3 months later at time point 3 (TP3) in March 2017. The survey was designed using the software package Survey Monkey©. At each TP, participants were invited by an email linked to the web-based survey. Participants with incomplete or missing responses to the survey were reminded after 1 week. Only participants answering all questions in the questionnaire were invited to participate at the following TP.

At TP1, participants were asked for demographic information, a subjective self-assessment of their familiarity with Standard Precautions, the University Hospital Zurich infection prevention and control concept (a collection IPC-relevant SOP on the intranet), and the SOP document “Standard precautions – the basics”. According to their randomization group, the participants were then asked to either watch the video, read the SOP, or neither of both. They were then asked to evaluate their satisfaction with the video/SOP, and to answer 32 knowledge assessment questions (Eight “scenarios” with four questions, each to be answered as correct or incorrect; Table 2). The questionnaire contained four questions whose content was not explicitly mentioned neither in the video nor the SOP. The content of another two questions was only covered by the SOP but not the video.

At TP2 and TP3, the participants were asked again for a subjective self-assessment of their familiarity with Standard Precautions, the University Hospital Zurich infection prevention control concept, and their exposure to either video or SOP since the last TP. Also, they had to re-evaluate their satisfaction with the video/SOP, and were asked the same eight knowledge assessment questions again. See Additional file 1 and 2 for an English translation of the questionnaires.

### Interventions

The teaching intervention in our study was to either watch the 5-min video “Welcome on board” or to read the SOP “Standard precautions – the basics”. For comparability reasons, time for reading the SOP was advised not to be longer than 5 min. Both the video and the SOP equally cover the six fundamental topics of Standard Precautions: hand hygiene, use of personal protective equipment (PPE), professional appearance, respiratory hygiene, aseptic technique, environmental cleaning, and device disposal and reprocessing.

### The “Welcome on board” video

“Welcome on board – Infection prevention at the University Hospital Zurich” is a 5-min video about Standard Precautions [https://www.youtube.com/watch?v=uHzwbdBoZVg]. The screenplay was written in collaboration of the IPC team and a film director. The video was filmed with a professional film team including a director, a director of photography, a gaffer, a sound engineer, a costume designer, a make-up artist, two professional actors and 15 extras. Hospital-known infection prevention team members took part in the video. Humor was emphasized as central feature of this “edutainment” video. The scenes were set inside an airplane with passengers appearing as patients. The plot was a “mash-up” between an in-flight safety video and infection prevention instructions. The audience witnesses a cabin crew/infection prevention team member giving instructions to a novice cabin crew member/healthcare provider.

### Standard operating procedure “Standard precautions – the Basics”

“Standard precautions – the Basics” is a three page, 600-word SOP, which was designed for high usability in iterative rounds of design and user testing. In the “Top-Section”, the main messages of the document are summarized. Then, the six topics are elaborated serially, in short and well-structured sections. For clarity and comprehensibility reasons, the word count of every section is reduced to the minimum. Where considered beneficial, the text is illustrated with iconic visualizations. The SOP has been accessible and promoted among all HCP on the University Hospital Zurich intranet website since June 2016 as part of all infection prevention and control SOPs (Additional file 3).

### Endpoints

The primary endpoint was the performance of the video group answering the eight multiple response knowledge assessment questions immediately after the intervention at TP1, compared to the SOP group and the no-intervention group. Secondary endpoints were the comparison of the performance of the video group to the other two groups one (TP2) and 3 months (TP3) after the intervention to test the long-term effect of the video, the assessment of the HCP’s satisfaction with the video and the SOP, and evaluation of independent predictors of ‘good knowledge’ about Standard Precautions.

### Statistical analysis

All employees responding to our invitation were included in this study. Variables were assessed within and between the assigned groups at and between the different TPs using non-parametric tests (Fisher’s exact test, Wilcoxon rank-sum test, Wilcoxon signed-rank test, Kruskal-Wallis test - where appropriate - with Dunn’s multiple pairwise comparison with Holm correction, or Friedman test). Predictive factors for higher scores on the knowledge assessment were analyzed using uni- and multivariable linear regression analysis. As an evaluation of the teaching methods, answers regarding satisfaction were measured using a six-point Likert-type scale and compared by using Student’s t-test with Welch approximation.

Sensitivity analyses for performance on the knowledge assessments were conducted including only participants answering the questionnaires at all three time points, participants who dropped out after TP1 or TP2, and excluding participants who were re-exposed to the video between TPs.

Statistical analysis was performed using Stata 15.1 SE (StataCorp, College Station, TX, USA).

## Results

**Fig. 1:**
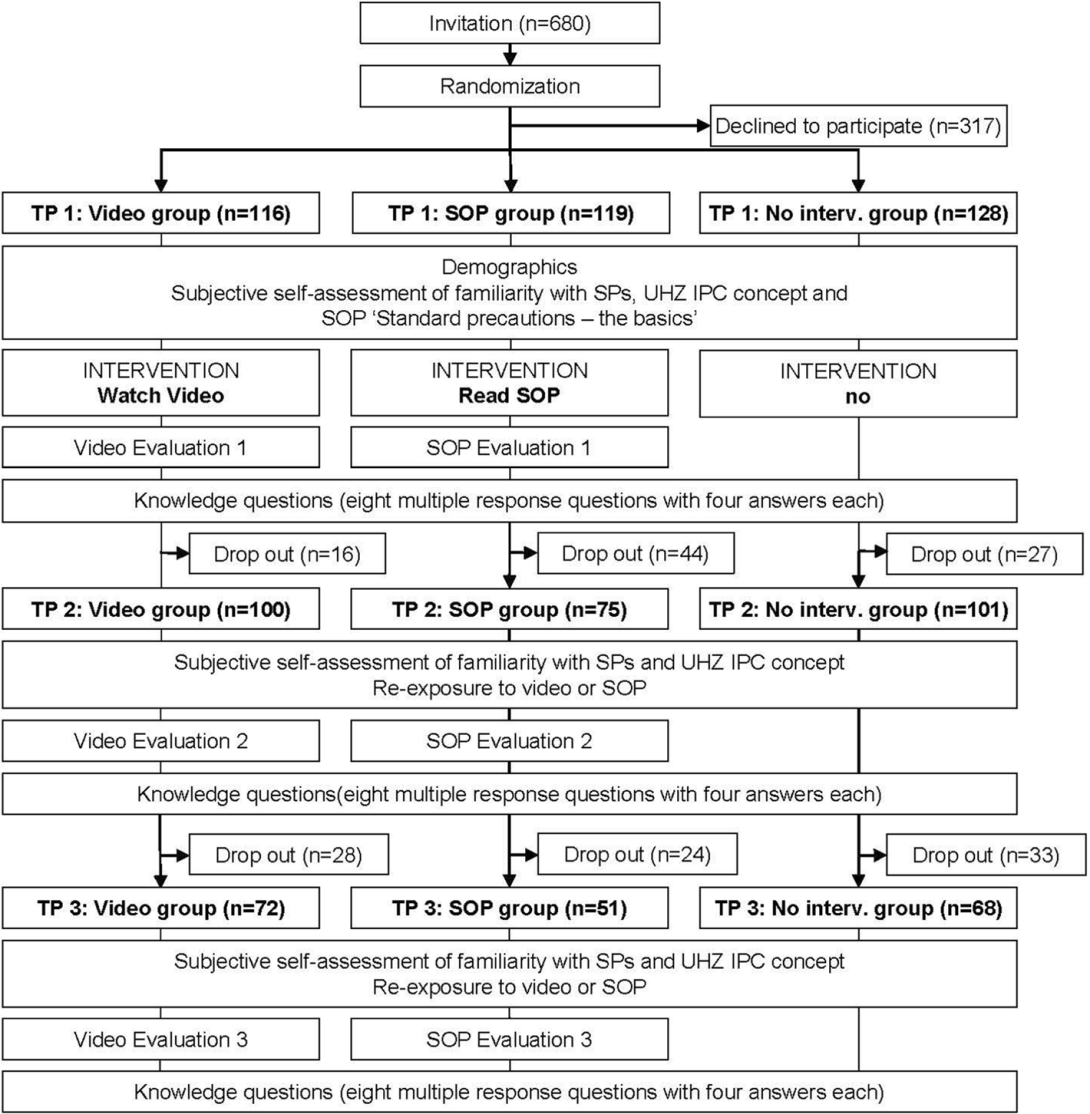
Study flow chart depicting interventions and questionnaires of all three study groups. Abbreviations: No Interv., no intervention; IPC, infection prevention and control; SOP, standard operating procedure; SP, standard precautions; UHZ, University Hospital Zurich

Of 680 invited HCP, 363 (53.4%) participated in the study at TP1. Thereof, 276 (76.0%) and 191 (52.6%) completed the questionnaires at TP2 and TP3, respectively. The dropout rate from TP1 → TP2 and TP1 → TP3 was 13.8 and 37.9% for the video group, 37.0 and 57.1% for the SOP group, and 21.1 and 46.9% for the no-intervention group, respectively. The study flowchart and the number of participants at each time point are illustrated in Fig. 1. Demographic data, self-assessment regarding familiarity with Standard Precautions, the University Hospital Zurich hygiene concept and the SOP “Standard precautions – the basics” are shown in Table 1.

**Table 1:** Participant characteristics

		All groups (*n* = 363)	Video group (*n* = 116)	SOP group (*n* = 119)	No-intervention group (*n* = 128)
Age in years, median (IQR)		34	34	33	33
		(28–42)	(28–42.5)	(28–41)	(28–42)
Female gender (%)		284 (78.2)	27 (76.7)	96 (80.7)	99 (77.3)
Profession (%)	Physician	91 (25.1)	25 (21.6)	34 (28.6)	32 (25)
	Nurse, nursing assistant, midwife	237 (65.3)	75 (64.7)	76 (63.9)	86 (67.2)
	Other profession (e.g. radiographer, therapist)	35 (9.6)	16 (13.8)	9 (7.6)	10 (7.8)
Professional experience (%)	< 1 year	29 (8.0)	13 (11.2)	7 (5.9)	9 (7)
	1–5 years	114 (31.4)	29 (25)	42 (35.3)	43 (33.6)
	5–10 years	69 (19.0)	24 (20.7)	25 (21.0)	20 (15.6)
	> 10 years	151 (41.6)	50 (43.1)	45 (37.8)	56 (43.8)
Duration of employment at the UHZ (%)	< 1 year	70 (19.3)	27 (23.3)	20 (16.8)	23 (18.0)
	1–5 years	156 (43.0)	42 (36.2)	60 (50.4)	54 (42.2)
	5–10 years	65 (17.9)	23 (19.8)	18 (15.1)	24 (18.8)
	> 10 years	72 (19.8)	24 (20.7)	21 (17.7)	27 (21.1)
Are you familiar with the term "Standard precautions"?	Yes, I’m very familiar	123 (33.9)	41 (35.3)	30 (33.6)	42 (32.8)
	I know some elements of standard precautions	163 (44.9)	55 (47.4)	51 (42.9)	57 (44.5)
	I can imagine what it is about	72 (19.8)	19 (16.4)	27 (22.7)	26 (20.3)
	Does not mean anything to me	5 (1.4)	1 (0.8)	1 (0.8)	3 (2.3)
Is familiar with the IPC concept of the UHZ and its SOP		307 (84.6)	101 (87.1)	103 (86.6)	103 (80.5)
Did read the SOP about “Standard precautions - the basics” (outside study setting)		178 (49)	60 (51.7)	57 (47.9)	61 (47.7)
Did read the entire SOP about “Standard precautions - the basics” (outside study setting)		53 (14.6)	21 (18.1)	16 (13.4)	16 (12.5)
Did read the SOP about “Standard precautions - the basics” more than once (outside study setting)		79 (21.8)	33 (28.4)	21 (17.6)	25 (19.5)
Did read the SOP about “Standard precautions - the basics” during the previous month (outside study setting)		70 (19.3)	26 (22.4)	24 (20.2)	20 (15.6)

*Abbreviations: IQR* Interquartile range, *SOP* Standard Operating Procedure, *UHZ* University Hospital Zurich

The participants were predominantly female (78.2%) with a median age of 34 years. The majority were nurses (65.3%), had a work experience of at least 5 years (60.6%), and considered themselves familiar with at least ‘some elements’ of Standard Precautions (78.8%). Through the randomization process, all 3 study groups were balanced on these demographic and professional characteristics.

### Effect of educational intervention on knowledge scores

**Fig. 2:**
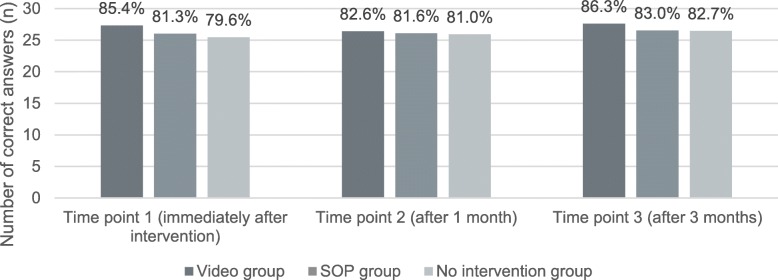
Mean number (y axis) and percentage (number above bars) of correct answers to the 32-item knowledge assessment questionnaire (by study group and time point) Abbreviations: SOP, standard operating procedure

Figure 2 shows the mean number and percentage of correct answers to the 8 × 4 knowledge questions for all study groups and time points. At all three TP, the video group scored best. At TP1, the video group, with a mean of 27.34 correct answers (85.4%), scored significantly better than the SOP group (26.03 correct answers (81.3%)), and the no-intervention group (25.48 correct answers (79.6%)) (*p* = .001). In the pairwise comparison, the SOP group also scored better than the no-intervention group (*p* = .006). At TP2, there was no difference between the three groups (*p* = .364). At 3 months follow-up (TP3), the video group again scored better (mean of 27.63 correct answers (86.3%)) than the SOP (mean of 26.55 correct answers (83.0%)) and the no-intervention group (mean of 26.47 correct answers (82.7%)) (*p* = .045).

Table 2 shows the results of the 8 × 4-item knowledge assessment questionnaire in detail, the number of correct answers, and the percentages of correct answers at TP1 per study group. Participants of the video group scored better than the SOP group and/or no-intervention group in questions concerning respiratory hygiene, PPE, and professional appearance (i.e. questions Q 2.4, Q 3.2–3.4, Q 4.2–4.3, Q 5.1, Q 6.1, Q 8.1, Q 8.3).

**Table 2:** Knowledge assessment with 32-item questionnaire at time point 1 (immediately after intervention)

	Question		Correct answer	Video group, percent correct answers	SOP group, percent correct answers	No-intervention group. Percent correct answers	*p*-value
Q 1.1.	Wearing non sterile gloves is indicated when	..drawing blood	right	94.8	96.6	95.3	.810
Q 1.2.		..touching a patient who is an MRSA carrier ^a)^	wrong	54.3	43.7	61.7	**.017**
Q 1.3.		..emptying an urinary bag	right	98.3	97.5	98.4	.897
Q 1.4		..washing a patients‘face ^b^	wrong	73.3	84	85.2	**.042**
Q 2.1.	Wearing a surgical mask is indicated when	..drawing blood from patients’ suffering from an influenza	right	60.3	55.5	68.8	.094
Q 2.2.		..providing personnel care to a patient with an open tuberculosis ^a^	wrong	50.9	38.7	46.1	.168
Q 2.3.		..suctioning an intubated patient	right	91.4	93.3	82.0	**.014**
Q 2.4		..providing personnel care to a patient suffering from a cough	right	78.5	59.7	46.1	**<.001**
Q 3.1.	When performing an open suctioning of a patient with a tracheostoma I have to wear the following personal protective equipment:	gloves	right	98.3	96.6	93.8	.204
Q 3.2.		surgical mask	right	96.6	94.1	82.0	**<.001**
Q 3.3.		cap	wrong	97.4	87.4	95.3	**.007**
Q 3.4		goggles	right	91.4	79.0	67.2	**<.001**
Q 4.1.	To prevent the spread of a respiratory virus when suffering from a common cold...	..I’m only allowed to come to work if I’m vaccinated against influenza ^a^	wrong	98.3	98.3	99.2	.744
Q 4.2.		..I am allowed to cough and sneeze into a handkerchief which I dispose of immediately	right	88.8	82.4	71.1	**.002**
Q 4.3.		..I am allowed to cough and sneeze into my elbow	right	89.7	78.2	56.3	**<.001**
Q 4.4		..I wear a surgical mask when in contact to my colleagues	right	94.8	89.9	95.3	.213
Q 5.1.	When wearing non-sterile gloves, I ...	..have to always disinfect my hands before donning gloves	right	82.8	58.0	71.1	**<.001**
Q 5.2.		..have to always disinfect my hands after doffing gloves	right	93.1	94.1	94.5	.926
Q 5.3.		..am allowed to inject medication without prior hand disinfection after touching the same patient	wrong	87.1	88.2	90.6	.668
Q 5.4		..have to doff the gloves as soon as possible to prevent the spread of germs	right	79.3	73.1	69.5	.211
Q 6.1.	I have to disinfect my hands …	..before touching the bed table of a patient ^b^	right	88.8	76.5	77.3	**.023**
Q 6.2.		..before shaking hands with a patient	right	95.7	97.5	96.1	.777
Q 6.3.		..between touching a patients‘shoulder and emptying his urinary bag ^b^	wrong	90.5	88.2	84.4	.352
Q 6.4		..between touching a patients‘shoulder and injecting insulin	right	81.9	84.0	84.4	.863
Q 7.1.	Check the correct answers concerning aseptic procedures	When performing an aseptic procedure, I always have to wear short sleeves	right	82.8	79.8	68.8	**.025**
Q 7.2.		When performing an aseptic procedure, I always have to wear sterile gloves	wrong	60.3	58.0	58.6	.937
Q 7.3.		Aseptic procedures prevent the contamination of clean surfaces and material	right	76.7	83.2	89.1	**.037**
Q 7.4		If I talk during aseptic procedures I need to wear a mask	right	75.0	73.1	66.4	.298
Q 8.1.	When in direct patient contact, I’m NOT allowed to wear …	..wedding ring	right	98.3	89.1	78.1	**<.001**
Q 8.2.		..pearl earrings	wrong	88.8	90.8	85.9	.504
Q 8.3.		..nail polish	right	99.1	96.6	93.0	**.038**
Q 8.4		..watch	right	97.4	98.3	96.9	.842

Caption: 32-item questionnaire with the percentage of correct answers by study group at time point 1

*Abbreviations: MRSA* Methicillin resistant *Staphylococcus aureus*, *SOP* Standard Operating Procedure, *Q* Question

^a^ Topics not or only rudimentary thematized in video and SOP. ^b^ Topics not or only rudimentary covered in the video but covered in the SOP

Results remained categorically equal when analyzing only drop-out participants, participants completing all three surveys, and excluding participants who were re-exposed to video (data not shown).

By using uni- and multivariable linear regression models, we found independent associations of higher knowledge scores at TP1 for female sex (*p* = .003), members of the video group (*p* < .001), and those participants who described themselves as being familiar with the University Hospital Zurich infection prevention and control concept (*p* = .032). Univariate and multivariate estimates are shown in Table 3.

**Table 3:** Predictive factors for higher knowledge scores at time point 1

	Univariable analysis		Multivariable analysis	
	Coefficient (95%CI)	*P* value	Coefficient (95%CI)	*P* value
Female gender	1.12 (0.39–1.85)	.003	1.07 (0.36–1.78)	.003
Study group		<.001		
- No-intervention group	Ref.		Ref.	
- Video group	1.86 (1.14–2.58)		1.81 (1.10–2.52)	<.001
- SOP group	0.55 (−0.17–1.27)		0.46 (−0.26–1.16)	.201
Familiarity with UHZ hygiene concept	1.15 (0.31–1.99)	.007	0.89 (0.08–1.70)	.032
Profession		.065		
- Physicians	Ref.			
- Nurses	0.85			
- Other professions	0.48			
Work experience (in years)		.471		
- < 1	Ref.			
- 1–5	0.11 (−1.32–1.10)			
- 5–10	0.55 (−0.73–1.84)			
- > 10	0.29 (−0.89–1.47)			
Working at UHZ (in years)		.086		
- < 1	Ref.			
- 1–5	0.10 (−0.73–0.93)			
- 5–10	1.12 (0.12–2.11)			
- > 10	0.45 (−0.52–1.42)			

*Abbreviations: CI* confidence interval, *UHZ* University Hospital Zurich, *Ref* Reference group, *SOP* standard operating procedure

### Satisfaction with teaching material

Table 4 shows the eight evaluation questions for the video and SOP groups at TP1. Both interventions were similarly rated regarding importance and structure of contents, and both were considered suitable teaching methods. The video was rated higher regarding enjoyment (i.e. ‘I enjoyed the video/SOP’), regarding entertainment value (i.e. video/SOP considered entertaining), and emotional impact. At TP2, more participants declared being ready to recommend the video than the SOP to colleagues (mean 3.02 ± 1.63 vs 2.57 ± 1.35, *p* = .049) (Additional file 4). Video group members thought they recalled more content information than the SOP-group and more often talked about the video than the other two groups’ participants. This association, however, was not statistically significant (Additional files 4 and 5**).** Compliance and engagement with Standard Precautions assessed by self-evaluation, were equally stimulated by both interventions.

**Table 4:** Participants satisfaction with their assigned teaching method at time point 1

		Mean Score (SD) ^a^		
Category	Question asked in questionnaire	Video group (*n* = 116)	SOP group (*n* = 119)	*p*-Value
Enjoyment	I enjoyed the video/SOP	5.24 ± 0.88	4.98 ± 0.77	.018
Relevance	The video/SOP contains relevant information for my daily work	5.23 ± 0.81	5.29 ± 0.82	.563
Pleasure	The video/SOP is pleasant to watch/read	5.31 ± 0.80	5.11 ± 0.84	.061
Memorization	I think that the content of the video/SOP will stick to my memory	5.06 ± 0.93	4.93 ± 0.78	.255
Structure	The content of the video/SOP is well structured	5.13 ± 0.72	5.07 ± 0.80	.531
Entertainment value	The video/SOP is entertaining	5.19 ± 0.97	3.66 ± 1.28	<.001
Emotional impact	The video/SOP touched me emotionally	3.50 ± 1.57	2.08 ± 1.32	<.001
Qualification for teaching	The video/SOP qualifies as a teaching aid	4.98 ± 0.93	4.76 + 0.98	.070

*Abbreviations: SOP* standard operating procedure, *SD* standard deviation

^a^ Mean based on 1–6 scale where 6 = “Strongly Agree” and 1 = “Strongly Disagree”

## Discussion

This randomized controlled study found that watching the edutainment video “Welcome on board”, combining educational and entertaining elements, resulted in higher knowledge scores in Standard Precautions than reading the SOP or no intervention, immediately after the interventions and at 3 months follow-up. Participants of the video group reported a higher satisfaction and recommended their teaching aid (i.e. edutainment video) more often to colleagues than participants from the SOP group.

Many of the elements of Standard Precautions seem to be well known to HCPs in our hospital, as the no-intervention group already scored high. Still, after watching the video “Welcome on board”, video group participants scored a mean of two points higher than participants of the no-intervention group. This difference reached statistical significance, and is, in our opinion, clinically relevant. Knowing all elements of Standard Precautions is essential for patient and HCP safety - every single item may make a difference during routine hospital work. The video group scored also higher than the group reading the SOP, demonstrating the superior effectiveness of watching the video compared to having purposefully read the SOP.

E-learning techniques teaching IPC topics have shown positive results. Other than our study, all of these studies feature interactivity [10–14, 16, 21], multimodal approaches including videos [10, 11, 13, 14, 16, 21], text [10, 14, 16, 21], pictures [10, 13, 16], or a variety of knowledge tests, games or quizzes [10–14, 21]. The usefulness of a ‘video only’ intervention was tested in patient education and showed improvement in retention of information [22], and increased short-term knowledge [23]. Also, video interventions were shown to be effective in modifying health behavior of patients [24]. Many edutainment videos on hand hygiene are available – and Lim et al. have considered them educationally useful by a non-validated scoring system [25]. To our knowledge, this study is the first to assess the learning effect of and satisfaction with a standalone edutainment video on IPC Standard Precautions.

The higher scoring of the video group was mainly driven by higher scores in 10 of the 32 knowledge questions. These questions dealt with topics prominently addressed in the video - almost exclusively through humor -, such as wearing PPE when suctioning a patient, compliance with cough etiquette, and professional appearance. Banas et al. stated in a review about humor in education, that appropriate humor attracts and sustains attention and produces a relaxed and productive learning environment [26]. Our findings are also in line with the results of Kaplan et al., who found that humor leads to a better retention of teaching material [27].

To assess the long-term effect of the video against the SOP and no intervention, we reapplied the same knowledge questions in a randomized sequence 1 and 3 months after the intervention. In the no-intervention and SOP groups, we saw an incremental increase in scores at consecutive TPs. This might be explained by the exposure to the questionnaire itself or by the increasing engagement of the participants with Standard Precautions, supported by the fact that roughly half of all participants declared being stimulated to use Standard Precautions by participating in our study (data not shown). The video group scored highest both 1 and 3 months after the intervention. Subjectively, the video participants tended to rate the video to better ‘sink into long-term memory’ than the SOP group participants and felt to remember more of the video elements after 1 month compared to the SOP elements. This suggests that the edutainment video might have a more favorable long-term effect than reading the SOP.

Educational videos are only useful if watched. Hypothesizing that higher satisfaction leads to higher utilization, we also assessed the participants’ satisfaction with the teaching material. Interestingly, both, the video and the SOP intervention, were rated positively. Still, the video was rated higher in terms of entertaining effect and appealing emotions, and was generally considered to be the better teaching aid compared to the SOP. More members of the video group talked to a colleague about the video or even recommended other HCPs to watch the video than SOP group members reading the SOP. Also, the fact that the video-group had the lowest dropout rate can be interpreted as a sign of relatedness and sustained attention to the topic. Still, if those who watched the video actually shared it with others, e.g. via social media, leading to a ‘viral’ spread among peers, has to be assessed in future studies.

Our study has limitations. First, participant blinding is not feasible in educational studies and recall bias may be unavoidable in this setting. Second, the knowledge assessment questionnaire contained four questions whose topics were not or only rudimentarily addressed neither in the video nor the SOP and two questions whose content was only addressed in the SOP and not the video. We included these questions with the argument that both the video and the SOP not only explicitly but also implicitly teach IPC topics. Still, we carefully minded to not include questions only covered in the video to not bias the results in favor of the video group. Third, drop-out rate was considerable, especially in the SOP group. Still, sensitivity analysis showed equal results when analyzing only drop-out participants or participants completing all three surveys, respectively. Last, and most importantly, better knowledge of Standard Precautions alone does not necessarily lead to higher adherence to Standard Precautions [28].

## Conclusion

We demonstrated that an edutainment video on Standard Precautions is not only entertaining but also educational by finding highest knowledge scores in the video group. Transporting the content in a humorous way seemed to lead to especially high scoring. The video was preferred over the SOP regarding emotional and entertainment values, and was more often recommended to colleagues. This study addressed the first two levels of Kirkpatrick’s “four-level model” for evaluating training and education programs, ‘learning effectiveness’ and ‘reactions of participants’; further research is needed to evaluate the effect of edutainment videos on ‘behavior’ and ‘impact on patients’ or HCWs’ health’.

## Additional files

Additional file 1: Study questionnaire time point 1 (immediately after intervention). (DOCX 24 kb)

Additional file 2: Study questionnaire time point 2 (1 month after intervention) and 3 (3 months after intervention). (DOCX 22 kb)

Additional file 3: Standardmassnahmen Übersicht. (DOCX 137 kb)

Additional file 4: Participants satisfaction with their assigned teaching method at time point 2. (DOCX 18 kb)

Additional file 5: Participants satisfaction with their assigned teaching method at time point 3. (DOCX 18 kb)

## Abbreviations

CDC: Centers for Disease Control and Prevention
CI: Confidence interval
HCP: Healthcare provider
IPC: Infection prevention and control
PPE: Personal protective equipment
SOP: Standard operating procedure
TP: Time point
