# Supplementary material for: Effectiveness of an edutainment video teaching standard precautions – a randomized controlled evaluation study
Source: Antimicrob Resist Infect Control. 2019 May 22;8:82. doi: 10.1186/s13756-019-0531-5 (PMC6530153; doi:10.1186/s13756-019-0531-5)
Supplement: Supplementary file 1 — Study questionnaire time point 1 (immediately after intervention). (DOCX 24 kb) [file 13756_2019_531_MOESM1_ESM.docx]

# Additional file 1 - Study questionnaire time point 1 (immediately after intervention)

**Part 1**

Are you familiar with the term „Standard precaution“?

Yes, I’m very familiar

I know some elements of standard precautions

‘It rings a bell’

Does not mean anything to me

Are you familiar with the infection prevention and control concept of the University Hospital Zurich and its SOP?

Yes  No

If yes, did you ever read the SOP “Standard precautions – the Basics”?

Yes  No

If you read the SOP “Standard precautions – the Basics”, did you fully read it?

Yes  No

If you read the SOP “Standard precautions – the Basics”, how many times did you read it?

Once

Twice

Three times

More than three times

If you read the SOP “Standard precautions – the Basics”, when was the last time you read it?

Less than one week ago

More than one week ago, but less than one month ago

More than one month ago

**Part 2** *[not visible to no-intervention group]*

Please assess the video/the SOP:

1. doesn’t apply at all
2. does not apply
3. does rather not apply
4. does rather apply
5. does apply
6. does completely apply

I enjoyed the [video] [SOP]

(1)  (2)  (3)  (4)  (5)  (6)

The [video] [SOP] contains relevant information for my daily work

(1)  (2)  (3)  (4)  (5)  (6)

The [video] [SOP] is pleasant to watch/read

(1)  (2)  (3)  (4)  (5)  (6)

I think, the content of the [video] [SOP] will stick to my memory

(1)  (2)  (3)  (4)  (5)  (6)

The content of the [video] [SOP] is well structured

(1)  (2)  (3)  (4)  (5)  (6)

The [video] [SOP] is entertaining

(1)  (2)  (3)  (4)  (5)  (6)

The [video] [SOP] touched me emotionally

(1)  (2)  (3)  (4)  (5)  (6)

The [video] [SOP] qualifies as a teaching aid

(1)  (2)  (3)  (4)  (5)  (6)

**Part 3**

*[The 32 skill questions are listed in* ***Table 2*** *of the main manuscript]*
