# Supplementary material for: Effectiveness of an edutainment video teaching standard precautions – a randomized controlled evaluation study
Source: Antimicrob Resist Infect Control. 2019 May 22;8:82. doi: 10.1186/s13756-019-0531-5 (PMC6530153; doi:10.1186/s13756-019-0531-5)
Supplement: Supplementary file 2 — Study questionnaire time point 2 (1 month after intervention) and 3 (3 months after intervention). (DOCX 22 kb) [file 13756_2019_531_MOESM2_ESM.docx]

# Additional file 2 - Study questionnaire time point 2 (one month after intervention) and 3 (three months after intervention)

**Part 1**

Are you familiar with the term „Standard precaution“?

Yes, I’m very familiar

I know some elements of standard precautions

‘It rings a bell’

Does not mean anything to me

Are you familiar with the infection prevention and control concept of the University Hospital Zurich and its SOPs?

Yes  No

**Part 2**

Since the last survey, did you read the SOP “Standard precautions – the Basics”?

Yes  No

Since the last survey, did you watch the video “Welcome on board”?

Yes  No

**Part 3**

Please assess the video/the SOP:

1. doesn’t apply at all
2. does not apply
3. does rather not apply
4. does rather apply
5. does apply
6. does completely apply

I can remember some of the elements of the [video] [SOP] well *[not visible to no-intervention group]*

(1)  (2)  (3)  (4)  (5)  (6)

I can remember ALL of the elements of the [video] [SOP] well *[not visible to no-intervention group]*

(1)  (2)  (3)  (4)  (5)  (6)

I did talk about the [video] [SOP] with my colleagues *[not visible to no-intervention group]*

(1)  (2)  (3)  (4)  (5)  (6)

I did recommend the [video] [SOP] to my colleagues *[not visible to no-intervention group]*

(1)  (2)  (3)  (4)  (5)  (6)

I dreamt of the [video] [SOP] *[not visible to no-intervention group]*

(1)  (2)  (3)  (4)  (5)  (6)

I transferred the content of the [video] [SOP] [questionnaire] to my everyday working life and judge my compliance with standard precautions to be better now

(1)  (2)  (3)  (4)  (5)  (6)

Answering the questionnaire animated me to engage myself with Standard Precautions

(1)  (2)  (3)  (4)  (5)  (6)

**Part 4**

*[The 32 skill questions are listed in* ***Table 2*** *of the main manuscript]*
