# Supplementary material for: Effectiveness of an edutainment video teaching standard precautions – a randomized controlled evaluation study
Source: Antimicrob Resist Infect Control. 2019 May 22;8:82. doi: 10.1186/s13756-019-0531-5 (PMC6530153; doi:10.1186/s13756-019-0531-5)
Supplement: Supplementary file 3 — Standardmassnahmen Übersicht. (DOCX 137 kb) [file 13756_2019_531_MOESM3_ESM.docx]

# Additional file 3 Standardmassnahmen Übersicht

| Das Wichtigste in Kürze | |
| --- | --- |
| **Standardmassnahmen** | Die wichtigsten Massnahmen in der Verhinderung von Übertragung von Erregern  Werden bei allen Patienten sowohl im ambulanten als auch im stationären Setting angewandt |

| Händehygiene | | |
| --- | --- | --- |
| [**Hygienische Händedesinfektion**](http://intranet.usz.ch/Documents/Hygienische%20H%C3%A4ndedesinfektion%20%20Hygieneordner_1000082969.pdf) | 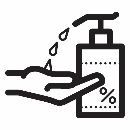 | 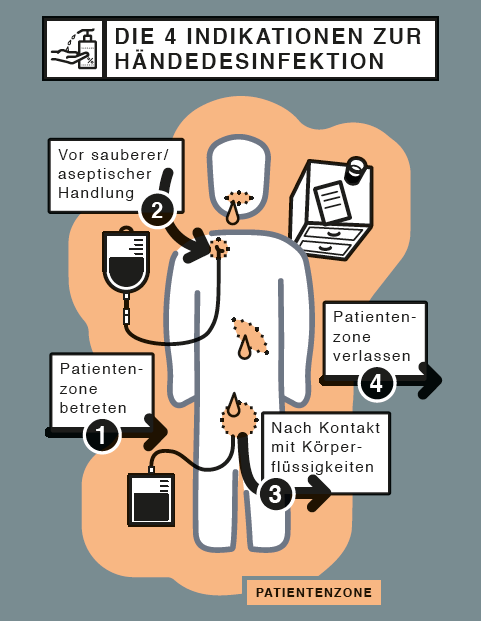   1. Patientenzone betreten 2. Vor sauberen/aseptischen Handlungen 3. Nach Kontakt mit Körperflüssigkeiten 4. Patientenzone verlassen |
| **Hände waschen** | 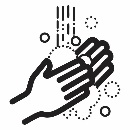 | Bei Arbeitsantritt und nach Arbeitsende  Bei sichtbarer Verschmutzung  Nach Toilettengang  Nach Kontakt mit *Clostridium difficile*, im Falle eines *C. difficile*-Outbreaks |
| **Hautschutz / Hautpflege** |  | Ziel: Intakte Haut der Hände  Hautschutzcrème vor Arbeitsbeginn und während der Arbeit  Hautpflegecrème regenerierend nach Arbeitsende |

| Kleidung und Schmuck - Im direkten Patientenkontakt, in Patientenumgebung und bei aseptischen Tätigkeiten | |
| --- | --- |
| **Dienstkleidung** | Täglich wechseln, bei Verschmutzung sofort wechseln  Private Kleidung muss mit Dienstkleidung bedeckt sein, Handgelenke müssen frei sein  Bei aseptischen Tätigkeiten: Keine langen Ärmel  Ausserhalb des Patientenzimmers sind Baumwolljacken (waschbar bei 60°) mit langen Ärmeln erlaubt |
| **Bereichskleidung** | Im Operationssaal, in der Zentralsterilisation, auf der Stammzell-Transplantationsstation und auf der Intensivstation für Brandverletzte gelten separate Regelungen |
| **Schmuck** | Erlaubt sind   - Kleine Ohrringe - Feine, kurze Halsketten   Nicht erlaubt sind   - Ringe (inkl. Ehering) - Armbanduhren, Armketten, Armbänder - Dermal Anchor und Piercing an Händen und Unterarmen |
| **Fingernägel** | Nicht erlaubt sind   - Nagellack - Lange Fingernägel - Künstliche Fingernägel |
| **Haare** | Lange Haare hochstecken  Mittellange Haare zusammenbinden |
| **Anderes** | Keine Schienen, Gips, Verbände an Unterarmen, Hand und Fingern (Ausnahme in Absprache mit dem personalärztlichen Dienst).  Begründung: Hände können nicht desinfiziert werden |

| Persönliche Schutzmassnahmen | | |
| --- | --- | --- |
| **Handschuhe unsteril** | 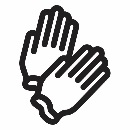 | Handschuhtragen mit der falschen Indikation erhöht das Übertragungsrisiko!  Indikationen:   - Bei voraussichtlichem Kontakt mit Körperflüssigkeiten - Bei Arbeiten mit chemischen Substanzen (Zytostatika, Flächen- und Instrumentendesinfektionsmittel)   Nach Ende der Handlung sofort ausziehen und die Hände desinfizieren |
| **Chirurgische Maske** | 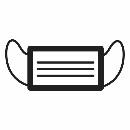 | Indikationen:   - Bei möglichen Spritzern von Körperflüssigkeiten, Zytostatika oder Desinfektionsmitteln ins Gesicht (z.B. offenes Absaugen, hustender Patient, Zubereitung von Desinfektionsmitteln etc.) - Bei aseptischen Tätigkeiten, wenn gesprochen wird (z.B. Verbandwechsel, Infusionen- und Injektionen richten) - Bei invasiven Tätigkeiten (z.B. Einlage ZVK, Lumbalpunktionen)   Entfernen der Maske sofort nach Gebrauch, bei sichtbarer Verschmutzung und bei Durchfeuchtung. |
| **Einwegschürze**  (Plastikschürze oder Einwegschürze „Iso“) | 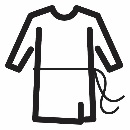 | Indikationen:   - Bei möglicher Durchfeuchtung der Berufskleider (z.B. bei Verbandwechsel, Körperpflege oder Arbeiten mit Desinfektionsmittel-Tauchbädern) - Bei sehr nahem Patientenkontakt (z.B. Beine des Patienten werden auf die Schulter des OP-Personals gelegt, Physiotherapeutin kniet auf das Bett, Baby wird auf Schulter getragen) |
| **Schutzbrille** | 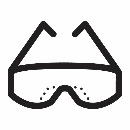 | Indikationen:   - Bei möglichen Spritzern von Körperflüssigkeiten (z.B. offenes Absaugen), Zytostatika oder Desinfektionsmittel ins Gesicht |
| **Haube** |  | Indikationen:   - Bei invasiven Tätigkeiten (Einlage ZVK, Herzkathetereinlage etc.), siehe entsprechende Richtlinien |

| [Hustenetikette für Mitarbeitende](http://intranet.usz.ch/Documents/Sch%C3%BCtzen%20Sie%20Ihr%20Umfeld%20vor%20respiratorischen%20Viren_1000059109.pdf) | |
| --- | --- |
| **Husten und Schnupfen** | Chirurgische Schutzmaske tragen im Kontakt mit Patienten und Mitarbeitenden sowie bei aseptischen Tätigkeiten  Husten und Niesen in ein Papiertaschentuch, dieses sofort nach Gebrauch entsorgen und Hände desinfizieren  Wenn kein Papiertaschentuch zur Verfügung steht, Husten und Niesen in die Armbeuge |
| **Grippesymptome mit Fieber** | Zu Hause bleiben |

| [Aseptische Arbeitstechnik](http://intranet.usz.ch/Documents/Aseptische%20Arbeitstechnik%20%20Hygieneordner_1000082632.pdf) | | |
| --- | --- | --- |
| **Händedesinfektion** | 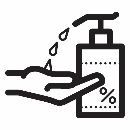 | Vor jeder aseptischen Tätigkeit |
| **Schutzmassnahmen** |  | Je nach Tätigkeit anziehen: sterile Handschuhe, chirurgische Maske, steriler Mantel, Haube |
| [**Hautdesinfektion**](http://intranet.usz.ch/Documents/Haut%20Schleimhaut%20und%20Wunddesinfektion%20%20Hygieneordner_1000082968.pdf) |  | Desinfizierte Stelle muss sichtbar feucht sein  Antiseptika eintrocknen lassen  Einwirkzeit beachten |
| **Non-Touch-Technik** |  | Steriles soll steril bleiben, deshalb nicht berühren!  Beispiele:   - Offene Systeme nicht an der sterilen Konnektionstelle berühren (Infusionssysteme) - Verbandwechsel mit Pinzette |
| **Steriles Material** |  | Verfalldatum und Unversehrtheit der Verpackung kontrollieren  Steriles Material so öffnen, dass eine Kontamination vom Inhalt ausgeschlossen werden kann  Hände und Arme nicht über sterile Tisch halten, Verpackungen nicht über sterilem Tisch öffnen |

| Aufbereitung und Entsorgung | |
| --- | --- |
| **Instrumente und Geräte** | Gezielte Desinfektion und Aufbereitung nach Gebrauch, siehe Aufbereitung im [Tauchbadverfahren](http://intranet.usz.ch/Documents/Aufbereitung%20im%20Tauchbadverfahren%20%20Hygieneordner_1000082993.pdf) und [Flächen- und Medizinprodukteaufbereitung](http://intranet.usz.ch/Documents/Fl%C3%A4chen%20und%20Medizinprodukteaufbereitung%20%20Hygieneordner_1000082996.pdf) |
| **Flächendesinfektion** | Gezielte Scheuer- Wischdesinfektion der Arbeitsfläche vor und nach Verrichtungen  Gezielte Desinfektion nach Verunreinigung mit Körperflüssigkeiten, siehe [Flächen- und Medizinprodukteaufbereitung](http://intranet.usz.ch/Documents/Fl%C3%A4chen%20und%20Medizinprodukteaufbereitung%20%20Hygieneordner_1000082996.pdf) |
| **Entsorgung medizinische Abfälle** | Siehe auch [Entsorgung medizinischer Sonderabfälle](http://intranet.usz.ch/Documents/Entsorgung%20medizinischer%20Abf%C3%A4lle_1000065301.pdf)   - Mit Körperflüssigkeiten verunreinigte, verletzungsgefährliche Gegenstände: Sharp-Behälter - Stark mit Körperflüssigkeiten verunreinigte Materialien: Sonderabfallbehälter 30 lt oder 50 lt |
