# Supplementary material for: Effectiveness of an edutainment video teaching standard precautions – a randomized controlled evaluation study
Source: Antimicrob Resist Infect Control. 2019 May 22;8:82. doi: 10.1186/s13756-019-0531-5 (PMC6530153; doi:10.1186/s13756-019-0531-5)
Supplement: Supplementary file 4 — Participants satisfaction with their assigned teaching method at time point 2. (DOCX 18 kb) [file 13756_2019_531_MOESM4_ESM.docx]

**Additional file 4 - Participants satisfaction with their assigned teaching method at time point 2**

|  | Mean Score (SD) * | | |
| --- | --- | --- | --- |
| **Question** | **Video group (n=100)** | **SOP group (n=75)** | **p-Value** |
| I can remember some of the elements of the film/SOP well (%) | 4.5 ± 0.82 | 4.27 ± 0.79 | .060 |
| I can remember ALL of the elements of the video/SOP well (%) | 3.32 ± 1.14 | 3.16 ± 1.00 | .327 |
| I did talk about the video/SOP with my colleagues (%) | 3.08 ± 1.62 | 2.67 ± 1.41 | .074 |
| I did recommend the video/SOP to my colleagues (%) | 3.02 ± 1.63 | 2.57 ± 1.35 | .049 |
| I dreamt of the video/SOP (%) | 1.31 ± 0.61 | 1.25 ± 0.62 | .548 |
| I transferred the content of the video/SOP to my everyday working life and judge my compliance with standard precautions to be better now (%) | 4.19 ± 1.03 | 4.37 ± 0.85 | .200 |
| Answering the questionnaire animated me to engage myself with the topic Standard Precautions (%) | 3.45 ± 1.31 | 3.67 ± 1.17 | .220 |
| I can remember some of the elements of the video/SOP well (%) | 4.5 ± 0.82 | 4.27 ± 0.79 | .060 |

* Mean based on 1–6 scale where 6 = “Strongly Agree” and 1 = “Strongly Disagree.”

Abbreviations: SOP, standard operating procedure; SD, standard deviation
