# Supplementary material for: Effectiveness of an edutainment video teaching standard precautions – a randomized controlled evaluation study
Source: Antimicrob Resist Infect Control. 2019 May 22;8:82. doi: 10.1186/s13756-019-0531-5 (PMC6530153; doi:10.1186/s13756-019-0531-5)
Supplement: Supplementary file 5 — Participants satisfaction with their assigned teaching method at time point 3. (DOCX 18 kb) [file 13756_2019_531_MOESM5_ESM.docx]

**Additional file 5 - Participants satisfaction with their assigned teaching method at time point 3**

|  | Mean Score (SD) * | | |
| --- | --- | --- | --- |
| **Question** | **Video group (n=72)** | **SOP group (n=51)** | **p-Value** |
| I can remember some of the elements of the film/SOP well (%) | 4.11 ± 1.12 | 4.08 ± 0.91 | .859 |
| I can remember ALL of the elements of the video/SOP well (%) | 2.86 ± 1.10 | 3.10 ± 0.98 | .241 |
| I did talk about the video/SOP with my colleagues (%) | 3.24 ±1.72 | 2.78 ± 1.39 | .111 |
| I did recommend the video/SOP to my colleagues (%) | 3.03 ± 1.75 | 2.78 ± 1.08 | .344 |
| I dreamt of the video/SOP (%) | 1.21 ± 0.47 | 1.22 ± 0.50 | .935 |
| I transferred the content of the video/SOP to my everyday working life and judge my compliance with standard precautions to be better now (%) | 3.96 ± 1.12 | 4.20 ± 1.11 | .247 |
| Answering the questionnaire animated me to engage myself with the topic Standard Precautions (%) | 3.26 ±1.09 | 3.16 ±1.10 | .595 |
| I can remember some of the elements of the video/SOP well (%) | 4.11 ± 1.12 | 4.08 ± 0.91 | .859 |

* Mean based on 1–6 scale where 6 = “Strongly Agree” and 1 = “Strongly Disagree.”

Abbreviations: SOP, standard operating procedure; SD, standard deviation
